# Comparisons of Early Outcomes Between the Superior Approach and Posterior Approach in Total Hip Arthroplasty for Patients With Osteonecrosis of the Femoral Head

**DOI:** 10.7759/cureus.97519

**Published:** 2025-11-22

**Authors:** Shine Tone, Yohei Naito, Gai Kobayashi, Akihiro Sudo, Masahiro Hasegawa

**Affiliations:** 1 Orthopaedic Surgery, Mie University Graduate School of Medicine, Tsu, JPN; 2 Orthopaedic Surgery, Yonaha Okanoue Hospital, Kuwana, JPN

**Keywords:** minimally invasive surgery, osteonecrosis of the femoral head, posterior approach, superior approach, total hip arthroplasty

## Abstract

Purpose

This study aimed to compare the early clinical outcomes between the superior approach (SA) and posterior approach (PA) in total hip arthroplasty (THA) for patients with osteonecrosis of the femoral head (ONFH), hypothesizing that SA offers advantages in terms of blood loss, complications, and early clinical outcomes.

Methods

A retrospective cohort study was conducted on 58 THAs performed on patients with ONFH between 2016 and 2023, divided into SA (n = 33) and PA (n = 25) groups. Demographic data, surgical parameters, radiographic outcomes, early clinical outcomes (Merle d’Aubigné-Postel and Harris Hip Scores (HHS)), and perioperative complications were analyzed. Statistical analyses included the Mann-Whitney U test, chi-square test, and Fisher’s exact test, with significance set at p < 0.05.

Results

The SA group demonstrated significantly lower intraoperative blood loss (251 ± 130.9 mL vs. 504 ± 631.5 mL; p < 0.01) and perioperative total blood loss (416.5 ± 264.5 mL vs. 694 ± 510.3 mL; p < 0.05) compared to that in the PA group. At 1.5 months, the SA group showed better ability to walk (Merle d’Aubigné-Postel score: 4.3 ± 1.4 vs. 3.4 ± 1.3; p < 0.05) compared to the PA group. While both groups exhibited significant postoperative improvements, no significant differences were observed in the HHS. Two cases of dislocation and one of nerve palsy were observed in the PA group, whereas no complications were reported in the SA group.

Conclusion

This SA offers a minimally invasive option with fewer complications and is particularly advantageous for patients with ONFH.

## Introduction

Total hip arthroplasty (THA) is an effective and reproducible surgical procedure that significantly reduces pain, improves the range of motion (ROM), and enhances the patient’s quality of life. However, complications associated with this procedure remain unavoidable. Correct implant placement and effective soft tissue management are critical for preventing complications and optimizing postoperative outcomes of THA [1,2]. Surgical approaches have been refined to minimize intraoperative soft tissue damage, facilitate postoperative recovery, and improve patient satisfaction [3,4]. Minimally invasive surgery (MIS)-THA has gained popularity, and various muscle- and tendon-sparing techniques have been introduced [5,6]. Although MIS-THA offers advantages over standard techniques, such as reduced pain, decreased intraoperative blood loss, and faster recovery, it is associated with a steep learning curve, which can result in a prolonged operative time and increased risk of perioperative complications [7,8].

Osteonecrosis of the femoral head (ONFH) is a serious condition that primarily affects middle-aged individuals. As the condition progresses, it often leads to collapse of the femoral head, ultimately necessitating THA [9]. The outcomes of THA in patients with ONFH remain controversial, with reports indicating higher complication and reoperation rates in this population, likely due to the younger age and higher activity levels of these patients [10]. A large cohort study conducted in Japan reported a relatively high dislocation rate (5.2%) after THA. The identified risk factors for dislocation include advanced age, obesity, use of the posterolateral approach, and a smaller implant head diameter [11]. While reports on MIS-THA for ONFH are increasing [12,13], studies focusing on MIS-THA using a superior approach are lacking.

This study aimed to compare the short-term clinical outcomes of THA using the superior approach with those of the conventional posterior approach in patients with ONFH. We hypothesized that the superior approach would have advantages in terms of blood loss, complications, and early clinical outcomes compared with the posterior approach.

## Materials and methods

Patient selection

**Figure 1:**
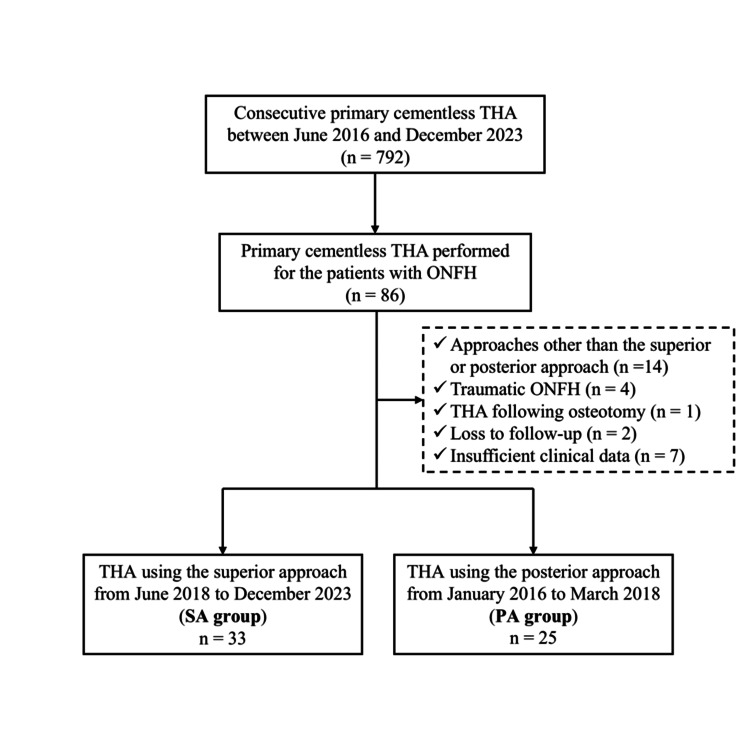
Patient flow diagram THA: total hip arthroplasty, ONFH: osteonecrosis of the femoral head.

The study was approved by the Institutional Review Board of Mie University Hospital (approval number H2018-083, approval date: April 3, 2018), and written informed consent was obtained from all participants. The study was conducted in accordance with the ethical standards of the 1964 Helsinki Declaration and its later amendments [14]. Between June 2016 and December 2023, 792 consecutive primary cementless THAs were performed at our hospital. The inclusion criterion was primary cementless THA performed for ONFH using either the SA or the PA in the lateral decubitus position. The exclusion criteria were THA performed using approaches other than the SA or PA, traumatic ONFH, THA after osteotomy, loss to follow-up, and insufficient clinical data. Ultimately, 58 THAs in 53 patients with ONFH were enrolled in this retrospective cohort study. Of these, 33 patients underwent surgery using the SA from June 2018 to June 2023, and the remaining 25 patients underwent surgery using the conventional PA from January 2016 to March 2018; thus, the two groups were divided according to the surgical period (Figure 1). In cementless acetabular cup implantation, the SQRUM TT Shell (Kyocera, Kyoto, Japan) was used in 18 hips, and the G7 PPS Finned Bone Master Acetabular Shell (Zimmer Biomet, Warsaw, IN, USA) was used in 15 hips in the SA group. In the PA group, the SQRUM TT Shell was used in seven hips, and the G7 PPS Finned Bone Master Acetabular Shell was used in 18 hips. In cementless stem implantation, the INITIA stem (Kyocera, Kyoto, Japan) was implanted in 18 hips, the Taperloc Microplasty stem (Zimmer Biomet, Warsaw, IN, USA) in 12 hips, and the Avenir stem (Zimmer Biomet, Warsaw, IN, USA) in three hips in the SA group. In the PA group, the INITIA stem was used in six hips, Taperloc Microplasty stem in 18 hips, and J-Taper stem (Kyocera, Kyoto, Japan) in one hip. All the patients provided informed consent to participate in this study. All THAs used a femoral head with a diameter of either 32 or 36 mm. Demographic data, including sex, age, body mass index, and classification and staging of ONFH, based on the Japanese Investigation Committee (JIC) [15] and Association Research Circulation Osseous (ARCO) [16] systems, were collected.

Surgical procedures

**Figure 2:**
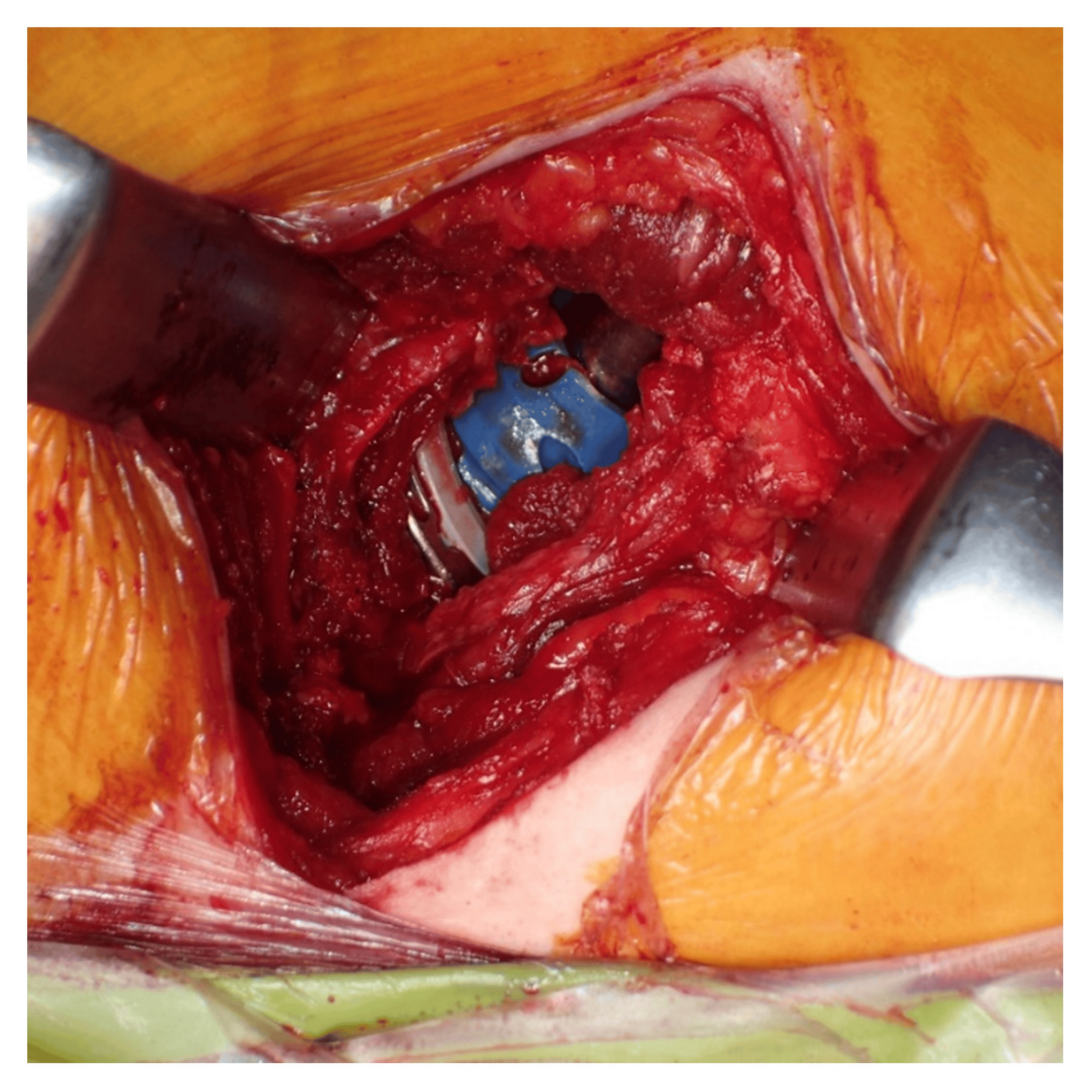
Post-implantation photograph

All the surgeries were performed by four THA specialists at a single institution. To maintain consistency in surgical techniques, implant selection, and intraoperative decision-making, a preoperative conference was held before all surgeries, and at least two of the certified arthroplasty surgeons participated in every operation. The SA was performed with the patient in the lateral decubitus position. The hip joint was positioned at 60° flexion, 30° adduction, and 25° internal rotation. A skin incision was made 6 cm proximal and 2 cm distal to the apex of the greater trochanter along the femoral axis. The gluteus maximus muscle was split in the direction of the muscle fibers, and the gluteus medius muscle was identified. By retracting the gluteus medius muscle anteriorly, the gluteus minimus muscle and piriformis tendon were identified. The gluteus minimus muscle was retracted anteriorly, the piriformis tendon was retracted posteriorly, and the capsule was exposed. The femoral neck and head were exposed through an incision in line with the course of the piriformis tendon from the acetabular rim of the trochanteric fossa. The retractors were placed inside the capsule around the anterior and posterior femoral necks. Femoral neck resection was performed using an oscillating saw, without dislocating the femoral head. The femoral head was then removed using a femoral head extractor. Dual-offset reamers are used to prepare the acetabulum. The acetabular components of all patients were placed using a navigation system. The cup orientation was planned to be 40° in radiographic inclination and 15° in radiographic anteversion relative to the functional pelvic plane [17]. The femur was subsequently prepared using broaches, and the femoral component was implanted with a target angle of 30° in stem anteversion relative to the posterior condylar axis, accounting for the native femoral anteversion of each patient. Dislocation repair was performed to confirm easy dislocation with no difference in leg length. A photograph obtained after implantation is shown in Figure 2.

**Figure 3:**
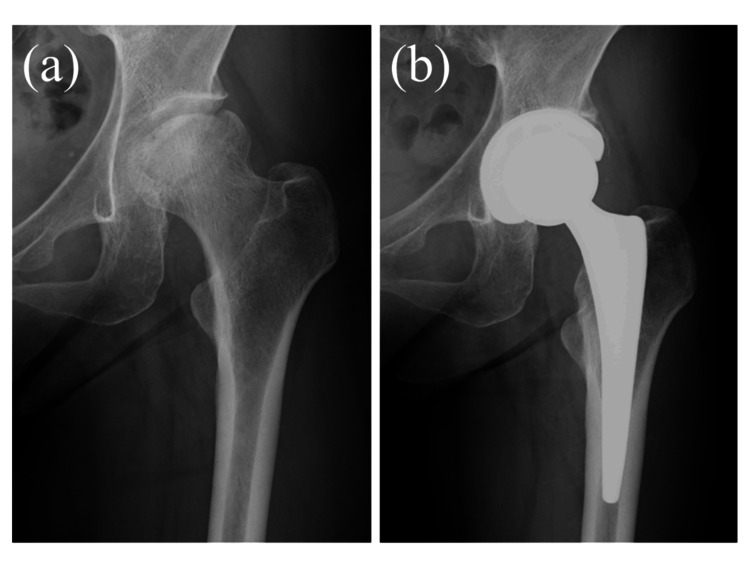
Preoperative anteroposterior radiograph (a) and postoperative anteroposterior radiograph at 12 months (b) of the superior approach for a patient with osteonecrosis of the femoral head

The hip joint capsule is anatomically closed. If the piriformis tendon had ruptured, it was repaired. Preoperative and postoperative anteroposterior radiographs of THA using the SA in patients with ONFH are shown in Figure 3.

The PA was performed with the patient in the lateral decubitus position. A conventional PA was used [18]. The released hip joint capsule and the short external rotator muscles were repaired.

Both groups received standardized perioperative care, including infection prophylaxis, venous thromboembolism prevention with Xa inhibitors, wound management, and functional rehabilitation. All patients underwent the rehabilitation protocol established at our hospital. Ambulation with full weight-bearing and active and passive ROM exercises were initiated on the first postoperative day. Dedicated physical therapists at our hospital provided identical rehabilitation programs for all patients, with daily sessions for two weeks after surgery.

Radiographic evaluation

All patients admitted to the study underwent preoperative and postoperative CT imaging to assess 3D component alignment. Postoperative CT images were performed two weeks after surgery using helical CT with metal artifact reduction, covering the area from the hip to the ankle with a 1-mm slice interval in all cases. Pre- and postoperative CT images were imported into the Zed Hip System (LEXI Co., Ltd., Tokyo, Japan), a validated CT-based 3D preoperative planning system for THA [19]. Cup radiographic inclination and anteversion were evaluated relative to the functional pelvic plane by the same independent observer in both groups. Stem anteversion relative to the posterior femoral condylar plane was assessed in both groups by an independent observer. The absolute values of the errors in radiographic inclination, radiographic anteversion, and stem anteversion were calculated by subtracting the postoperative angles from the target angles. The target angles were 40° for radiographic inclination, 15° for radiographic anteversion, and 30° for stem anteversion. Percentages within ±5° and ±10° from the target angles in radiographic inclination, radiographic anteversion, and stem anteversion were also analyzed.

Clinical evaluation

Both groups were clinically evaluated using the Merle d’Aubigné-Postel Hip Score [20] and Harris Hip Score (HHS) [21], with assessments performed preoperatively and 1.5, 3, 6, and 12 months postoperatively. The Merle d’Aubigné-Postel Hip Score was used, with a maximum of 6 points for pain, ROM, and gait function, such that a total of 18 points indicated full function. The surgical time was recorded from the initial incision to the completion of wound closure. The intraoperative blood loss, number of blood transfusions, and perioperative total blood loss were also evaluated. Transfusion criteria included a postoperative hemoglobin level < 7 g/dL or the presence of symptoms of tissue hypoperfusion, even if the hemoglobin level was >7 g/dL. Perioperative total blood loss was calculated indirectly from the change in hematocrit before surgery and on the first postoperative day according to Gross's formula [22]. The circulating blood volume was estimated using Nadler's formula [23]. Perioperative and postoperative complications, including infection, dislocation, intraoperative fracture, stem subsidence, and nerve palsy, were also recorded.

Statistical analysis

The primary outcome was the comparison of early clinical outcomes between the SA and PA up to one year postoperatively, assessed using the Merle d’Aubigné and Postel scoring system (pain, mobility, ability to walk, and total score) and the HHS. A previous study comparing clinical outcomes of different approaches reported mean HHS values of 88.4 ± 4.3 and 83.8 ± 6.0 at three months postoperatively, respectively [24]. Based on these results, power analysis was performed to calculate the sample size required to detect a mean difference of 4.6, assuming a standard deviation of 5.2. Using the Mann-Whitney U test, with a power of 0.8 and a two-tailed significance level of α = 0.05, the required sample size per group was determined to be at least 21 cases. All statistical analyses were performed using EZR (Saitama Medical Center, Jichi Medical University, Saitama, Japan), a graphical user interface for R (R Foundation for Statistical Computing, Vienna, Austria). The mean of postoperative cup radiographic inclination, radiographic anteversion, and stem anteversion was compared between the two groups. The operative time, intraoperative blood loss, number of blood transfusions patients, perioperative total blood loss, and complications were compared between the two groups. The Merle d’Aubigné and Postel scoring system (pain, mobility, ability to walk, and total score) and HHS at 1.5, 3, 6, and 12 months postoperatively were compared between the two groups. Statistical analyses were conducted using the Mann-Whitney U test for continuous variables, as all continuous data were non-normally distributed. The chi-square test and Fisher’s exact test were used to analyze categorical variables. Statistical significance was set at p < 0.05.

## Results

The patient demographics are listed in Table 1. No significant differences were observed between the two groups in the preoperative data.

**Table 1:** Demographic characteristics of the patients comparing the superior and posterior approaches SA: superior approach, PA: posterior approach, JIC: Japanese Investigation Committee, ARCO: Association Research Circulation Osseous. Data are presented as mean ± standard deviation, and categorical variables as numbers and percentages. p < 0.05 was considered statistically significant. ^a^Fisher’s exact test. ^b^Mann-Whitney U test.

	SA group (n = 33)	PA group (n = 25)	U-value	p-value
Sex				
Male-to-female ratio	23:10	12:13		0.11^a^
Age (years)	51.6 ± 13.4	54.0 ± 13.4	364	0.45^b^
Body mass index (kg/m^2^)	22.4 ± 4.4	23.8 ± 3.8	304	0.09^b^
Steroid use rate (n, %)	21, 63.6	17, 68.0		0.79^a^
JIC classification				
Type C1:C2	1:32	0:25		1^a^
JIC staging				
Stage 3A:3B:4	2:17:14	3:11:11		1^a^
ARCO classification				
Type 2:3	1:32	0:25		1^a^
ARCO staging				
Stage ⅢB:Ⅳ	19:14	14:11		1^a^

No significant differences were found between the two groups in the mean postoperative cup radiographic inclination, postoperative cup radiographic anteversion, and postoperative stem anteversion (Table 2).

**Table 2:** Postoperative radiographic evaluation comparing the superior and posterior approaches SA: superior approach, PA: posterior approach. Data are presented as mean ± standard deviation or numbers. p < 0.05 was considered statistically significant. ^a^Fisher’s exact test. ^b^Mann-Whitney U test.

	SA group (n = 33)	PA group (n = 25)	U-value	p-value
Mean value of postoperative				
Radiographic inclination (°)	40.9 ± 5.0	39.8 ± 5.7	465.5	0.26^b^
Radiographic anteversion (°)	18.7 ± 4.8	16.4 ± 8.9	472	0.22^b^
Stem anteversion (°)	30.4 ± 8.9	26.1 ± 9.3	490.5	0.13^b^
Absolute value of errors between postoperative and target angles				
Radiographic inclination (°)	3.8 ± 3.4	3.9 ± 4.1	390	0.93^b^
Radiographic anteversion (°)	4.4 ± 4.1	6.8 ± 5.7	286	0.08^b^
Stem anteversion (°)	6.7 ± 5.7	6.6 ± 7.6	437.5	0.7^b^
Percentage within ±5° from target angles				
Radiographic inclination (n, %)	26, 78.7	20, 80.0		1^a^
Radiographic anteversion (n, %)	22, 66.6	14, 56.0		0.43^a^
Stem anteversion (n, %)	17, 51.5	15, 60.0		0.6^a^
Percentage within ±10° from target angles				
Radiographic inclination (n, %)	31, 93.9	24, 96.0		1^a^
Radiographic anteversion (n, %)	31, 93.9	20, 80.0		0.22^a^
Stem anteversion (n, %)	24, 72.7	19, 76.0		1^a^

Although there were no significant differences in the operative time between the two groups, the mean intraoperative blood loss and perioperative total blood loss were significantly lower in the SA group than in the PA group (Table 3).

**Table 3:** Operative time, number of blood transfusion patients, intraoperative blood loss, and perioperative total blood loss comparing the superior and posterior approaches SA: superior approach, PA: posterior approach. Data are presented as mean ± standard deviation, and categorical variables as numbers and percentages. p < 0.05 was considered statistically significant. ^a^Fisher’s exact test. ^b^Mann-Whitney U test.

	SA group (n = 33)	PA group (n = 25)	U-value	p-value
Operative time (min)	134.9 ± 18.4	143.0 ± 31.9	322	0.16^b^
Number of blood transfusion patients (n, %)	2, 6.1	3, 12.0		0.64^a^
Intraoperative blood loss (mL)	251 ± 130.9	504 ± 631.5	238	< 0.05^b^
Perioperative total blood loss (mL)	416.5 ± 264.5	694 ± 510.3	283	< 0.05^b^

Both the Merle d’Aubigné and Postel scoring systems and the HHS showed significant postoperative improvement at all observation time points compared with the preoperative scores in both groups. No significant differences were observed in the preoperative Merle d’Aubigné and Postel scores between the two groups. However, 1.5 months postoperatively, the ability to walk score was significantly higher in the SA group than in the PA group. Although the total scores of the Merle d’Aubigné and Postel systems and the HHS postoperatively tended to be higher in the SA group than in the PA group, the differences were not statistically significant (Table 4).

**Table 4:** Perioperative outcomes comparing the superior and posterior approaches SA: superior approach, PA: posterior approach. Data are presented as mean ± standard deviation. p < 0.05 was considered statistically significant. ^a^Mann-Whitney U test.

	SA group (n = 33)	PA group (n = 25)	U-value	p-value
Merle d’Aubigné and Postel scoring system				
Preoperative				
Pain	2.6 ± 1.7	2.0 ± 1.6	496.5	0.17^a^
Mobility	4.6 ± 0.8	4.4 ± 0.8	465	0.38^a^
Ability to walk	3.2 ± 1.2	2.8 ± 0.8	489	0.21^a^
Total score	10.3 ± 2.7	9.2 ± 2.1	522	0.08^a^
Postoperative at 1.5 months				
Pain	5.4 ± 1.0	5.4 ± 0.9	432.5	0.73^a^
Mobility	5.7 ± 0.7	5.6 ± 0.6	448	0.47^a^
Ability to walk	4.3 ± 1.4	3.4 ± 1.3	544	< 0.05^a^
Total score	15.4 ± 2.2	14.4 ± 2.0	530.5	0.07^a^
Postoperative at 3 months				
Pain	5.5 ± 0.9	5.4 ± 1.0	452.5	0.46^a^
Mobility	5.8 ± 0.4	5.7 ± 0.6	462.5	0.24^a^
Ability to walk	4.7 ± 1.3	4.1 ± 1.4	526	0.06^a^
Total score	16.1 ± 2.0	15.2 ± 1.8	532	0.06^a^
Postoperative at 6 months				
Pain	5.6 ± 0.8	5.8 ± 0.8	347.5	0.33^a^
Mobility	5.9 ± 0.3	5.8 ± 0.5	398	0.76^a^
Ability to walk	5.0 ± 1.2	4.7 ± 1.3	435	0.41^a^
Total score	16.5 ± 1.9	16.3 ± 1.9	421	0.57^a^
Postoperative at 12 months				
Pain	5.8 ± 0.5	5.8 ± 0.4	446	0.45^a^
Mobility	6.0 ± 0.2	5.9 ± 0.4	433.5	0.4^a^
Ability to walk	5.2 ± 1.1	4.8 ± 1.1	525	0.06^a^
Total score	17.0 ± 1.4	16.4 ± 1.4	529	0.05^a^
Harris Hip Score				
Preoperative	49.9 ± 15.2	48.3 ± 19.1	448.5	0.58^a^
Postoperative at 1.5 months	82.4 ± 12.3	77.7 ± 9.6	492.5	0.06^a^
Postoperative at 3 months	88.4 ± 10.3	83.9 ± 9.5	468	0.07^a^
Postoperative at 6 months	90.5 ± 9.1	87.5 ± 9.8	466	0.2^a^
Postoperative at 12 months	92.9 ± 9.2	89.8 ± 9.0	478	0.1^a^

Regarding perioperative complications, two cases of dislocation and one case of nerve palsy were observed in the PA group, whereas no complications were reported in the SA group (Table 5).

**Table 5:** Perioperative and postoperative complications comparing the superior and posterior approaches SA: superior approach, PA: posterior approach. Categorical variables are presented as numbers and percentages. p < 0.05 was considered statistically significant. ^a^Fisher’s exact test.

	SA group (n = 33)	PA group (n = 25)	p-value
Intraoperative fracture (n, %)	0, 0	0, 0	1^a^
Dislocation (n, %)	0, 0	2, 8.0	0.18^a^
Infection (n, %)	0, 0	0, 0	1^a^
Stem subsidence (n, %)	0, 0	0, 0	1^a^
Nerve palsy (n, %)	0, 0	1, 4.0	0.43^a^

## Discussion

The most important finding of this study was that THA using the SA in patients with ONFH was associated with significantly lower intra- and perioperative total blood loss than THA using the PA. Additionally, patients who underwent the SA demonstrated significantly improved walking ability at 1.5 months postoperatively compared to those who underwent the PA. Notably, no postoperative complications were reported in patients who underwent surgery using the SA during the study period. These findings suggest that the SA may represent a viable and advantageous surgical option for relatively younger patients with ONFH who are at a higher risk of dislocation.

The SA was first introduced in 2004 by Murphy as a superior capsulotomy involving release of the piriformis and removal of the joint capsule to access the hip [25]. Subsequently, Capuano et al. reported the tissue-sparing posterior superior (TSPS) approach, a modification of the SA that does not require piriformis release or joint capsule resection [26]. The primary advantage of the SA lies in preserving the short external rotators, including the conjoint tendon and obturator externus, which enhance resistance to dislocation. In the largest reported series of SAs involving 1,454 consecutive THA procedures, the dislocation rate was remarkably low (0.21%) [27]. Additionally, the SA has been associated with less blood loss and a shorter length of hospital stay than the PA [28,29]. This benefit is attributed to the avoidance of iatrogenic injury to the medial circumflex femoral artery, a complication frequently encountered during the transection of external rotators using the PA. In the present study, no postoperative dislocations were observed when the SA was used. Additionally, the intraoperative and perioperative total blood loss was significantly lower than that in the PA. These findings suggest that a SA may be useful in reducing the risk of perioperative complications in patients with ONFH undergoing THA.

However, a notable challenge associated with the SA is the difficulty in achieving sufficient space for acetabular preparation, which can complicate cup placement. Navigation systems are recommended to ensure accurate cup positioning [30]. In this study, the use of navigation resulted in a 94% rate of cup placements within ±10° from the target angles in the SA, comparable to the accuracy achieved in the PA. In addition, specialized 45° offset reamers were initially required to address the challenges of acetabular preparation [25]. However, recent advancements have enabled the use of dual-offset reamers, as reported in the literature [26]. In this study, dual-offset reamers were used in all cases, eliminating the need for specialized surgical instruments. This adaptation allows the use of standard instruments from any implant manufacturer, making the SA more accessible and versatile for surgical teams.

Some studies have compared SAs with other approaches with respect to clinical outcomes. Murphy reported significantly higher Merle d’Aubigné and Postel system at 1.5 and 3 months postoperatively for the SA than for the lateral approach [25]. Capuano et al. reported significantly higher HHS at 1, 3, and 12 months and Western Ontario and McMaster Universities Osteoarthritis Index (WOMAC) scores at 3 months postoperatively for the SA than for the PA [26]. Romagnoli et al. reported that a SA was the only significant predictor of higher WOMAC scores. However, they noted no statistically significant differences in HHS or WOMAC scores between the SA and PA four months postoperatively [29]. In our study, a significant difference was observed in the ability to walk for 1.5 months postoperatively. However, both the total scores of the Merle d’Aubigné and Postel systems and the HHS tended to be higher in the SA than in the PA, although these differences were not statistically significant. These results indicate that the SA offers benefits in terms of early postoperative functional outcomes and bleeding risk compared with the posterior approach; however, unfortunately, there was no clear advantage in clinical outcomes. Further large-scale studies are required to validate these findings.

This study had several limitations. First, the sample size was small, which may have limited the generalizability of the results. A larger sample size is necessary to strengthen the statistical power and validate the findings in broader populations. Second, as a retrospective study, this study was susceptible to selection bias owing to the nonrandomized nature of the patient cohorts. However, the adoption of identical eligibility criteria for both cohorts likely minimized the risk of selection bias. Third, the types of implants used differed among the groups, which may have influenced the outcomes independently of the surgical approach. However, this variation reflected the actual clinical practice during the study period, and thus, the influence on the results is considered limited, although it remains a potential source of bias. Fourth, this study exclusively used the PA in the control group. Therefore, it remains unclear whether the SA offers greater utility than surgical approaches other than the PA. Fourth, the follow-up period in this study was limited to one year postoperatively, which precludes drawing definitive conclusions regarding mid- to long-term clinical outcomes and implant survival rates. Further investigations with longer follow-up periods are essential to evaluate these aspects comprehensively.

## Conclusions

This study compared the early clinical outcomes between the SA and PA in THA for patients with ONFH. THA using the SA for patients with ONFH appears to be a reliable and minimally invasive procedure, as it allows for better early postoperative functional outcomes and lower blood loss than the PA. However, further evaluation is needed to clarify its long-term outcomes.
